# Serum IGF-1, IGFBP-3 and their ratio: Potential biochemical growth maturity indicators

**DOI:** 10.1186/s40510-017-0165-1

**Published:** 2017-05-01

**Authors:** Nimisha Jain, Tulika Tripathi, S. K. Gupta, Priyank Rai, Anup Kanase, Shilpa Kalra

**Affiliations:** 1 New Delhi, India; 2 Department of Orthodontics and Dentofacial Orthopaedics, Maulana Azad Institute of Dental Sciences, MAMC Complex, BSZ Road, New Delhi, India; 3 Department of Biochemistry, Maulana Azad Medical College, New Delhi, India

**Keywords:** IGF-1, IGFBP-3, Biochemical growth marker

## Abstract

**Background:**

Determination of skeletal maturation and remaining growth potential is an essential part of treatment planning in orthodontics. The aim of our study was to determine the relationship between IGF-1 levels, IGFBP-3 levels with CVM staging to track the pre pubertal and pubertal growth spurts in female patients in North Indian population.

**Methods:**

This cross-sectional study was conducted on ninety female subjects in the age group of 8-20 years. Blood samples were collected and centrifuged and serum samples were then analysed by Human IGF-1 and IGFBP-3 enzyme-linked immunosorbent assay kits, specific for IGF-1 and IGFBP-3, respectively. CVM staging on lateral cephalometric radiograph was determined for all patients. Analysis of variance test followed by a post hoc test was used to compare mean IGF-1 and IGFBP-3 corresponding to six stages of cervical vertebrae maturation stages. Linear Pearson’s correlations were performed to determine the trends of IGF-1, IGFBP-3, and its ratio relating to CVM stage. The kappa statistic was used to measure inter and intra examiner reliability. *P* value <0.05 was considered as statistically significant.

**Results:**

Mean serum IGF-1 levels were found to be highest (403.3 ± 12.3 ng/ml) at CVMI3 stage of CVMI. The post-hoc test revealed a significant difference in IGF-1 levels between all stages of CVMI, thereby indicating a specific range of IGF-1 levels for a specific skeletal stage. Mean serum IGFBP-3 levels were found to be highest (5186.8 ± 1384.2 ng/ml) at CVMI4 stage of CVMI. The mean serum IGFBP-3 levels at CVMI4 were found to be significantly higher than the levels at all other CVMI stages except CVMI3 stage.

**Conclusions:**

IGF-1 and IGFBP-3 can serve as a potential biochemical indicator for assessment of skeletal maturity.

## Background

Determination of timing and amount of remaining craniofacial growth is an essential part of treatment planning in orthodonti CVMI and dentofacial orthopedi CVMI. Hence, assessment of maturation level and growth potential has become an integral part of diagnosis in our field. Biologic maturation and remaining growth potential has been determined by various methods [1–6]. Chronologic age, peak height velocity, and physical characteristic changes are highly unreliable method to predict pubertal growth spurt [7, 8].

Handwrist radiographs are considered to be highly reliable skeletal maturity indicator [9–13] but possess an additional radiation exposure hazard. Lateral cephalometric radiographs being commonly used in orthodontic diagnosis are popularly used to assess cervical vertebrae maturity stage of any individual for assessment of the level of pre pubertal/pubertal growth spurt [14–18]. Complexity in identification of landmarks and subjectivity of staging the x-rays are an inherent disadvantage of cervical vertebrae maturation (CVM) and hand wrist radiographs [19]. Also, because of the radiation exposure involved with these two methods, x-rays cannot be performed more than once a year.

Growth hormone has long been known to play a crucial role in in vivo linear growth. An increase in growth hormone play a role in the increased growth at puberty [20]. IGF-1 (Insulin like growth factor-1), a polypeptide hormone, is considered to be a mediator of growth hormone function. IGF-1 stimulates growth locally as well as systemically. An increase in level of IGF-1 has been correlated with pubertal growth spurt [21]. IGF-1 levels have been proposed as an alternative method to detect pubertal growth spurt timing in comparison to Cervical Vertebrae Maturity Index (CVMI) staging in different populations. Masoud et al [19] showed a positive correlation of blood spot IGF-1 levels to cervical vertebral maturity from prepubertal to late pubertal stages and a negative correlation with increasing time since onset of puberty in 83 (44 females and 39 males) subjects from Saudi population. Ishaq et al. [22] conducted a study on 120 subjects (60 females and 60 males) in circumpubertal age group from Egyptian population and compared the IGF-1 levels at different CVM stages. They observed the highest mean IGF-1 levels in stage 4 of cervical maturation in males and in stage three in females.

Also, IGF-1 levels have been correlated with hand wrist skeletal maturation pattern by Masoud et al. [23]. A marked positive correlation was observed between IGF-1 levels and hand-wrist stages from prepubertal stages to the stages of highest velocity of mandibular growth, whereas there was a moderate negative correlation between IGF-1 levels and hand-wrist radiograph stages from the levels associated with peak mandibular growth to the final hand-wrist stages.

Circulating IGF-1 is bound to specific Insulin like growth factor binding protein (IGFBPs) of which IGFBP-three binds the majority of IGFs. IGFBP-3 levels are regulated by growth hormone and have been suggested to provide additional information on growth hormone secretory capacity compared to IGF-1. Studies have shown that IGFBP-3 levels increase with age in children, with maximal levels in puberty [24]. However, the diagnostic value of IGFBP-3 is still controversial, perhaps because quality of normative data for IGFBP-3 varies.

A highly significant correlation between IGF-1 and IGFBP-3 on a molar basis (*r* = 0.84; *P* < 0.0001) has been revealed. Thus, it is speculated that IGFBP-3 is pivotal for circulating IGF bioactivity and that the increase in molar ratio between IGF-1 and IGFBP-3 reflects an increase in free, biologically active IGF-1 [24]. Blum et al. reported IGFBP-3 as a more accurate discriminator of GH dependent parameters than IGF-1 in IGF generation tests [25].

The aim of the present study was to know a relationship between IGF-1 levels, IGFBP-3 levels, and CVM staging to track the pre-pubertal and pubertal growth spurts in female patients in North Indian population.

## Methods

A cross sectional study was conducted on 90 female subjects in the age group of 8–20 years, selected from the outpatient department of orthodontics and dentofacial orthopedics of our institute. Sample size of 90 was calculated with *α* level of 0.05 and *β* power of 0.8 for this study. All the subjects were either patients under active treatment or new patients who required orthodontic treatment. For uniformity of the sample, only female subjects were included in the study. Patients with history of any chronic illness, growth abnormality and/or any bleeding disorder were excluded from the study.

A detailed history with personal information such as weight and height, medical history and family history was obtained to rule out the exclusion criteria. No additional blood investigations were carried out. The parents and subjects were informed about the research plan for approval for taking the blood sample and using the lateral cephalometric radiograph (which were taken for routine orthodontic examination) for the study. Ethical approval was obtained from the research ethical committee of the institute.

Lateral cephalometric radiograph was obtained. All the lateral cephalometric radiographs were taken by the same operator on the same machine in natural head position. The area of cervical vertebrae was traced on matte acetate paper by one examiner. The criteria of Hassel and Farman [17] were used to evaluate cervical vertebral radiographic morphology and accordingly the subjects were staged. All the subjects were grouped into six groups according to CVMI staging.

Blood samples were collected within a week after obtaining lateral cephalometric radiographs from the subjects for analysis of IGF-1 and IGFBP-3 by using red plain blood collection vials (Vacutech). Unlike Growth hormone, levels of IGF-1 do not fluctuate throughout the day. 2.5 ml of blood was drawn in the morning hours between 9 AM to 11 AM. Samples were centrifuged to separate serum and serum samples were then stored in sealed plastic container in a freezer at −70 °C. The samples were kept for not more than 4 months and were then analyzed by RayBio® Human IGF-1 and IGFBP-3 Enzyme-Linked Immunosorbent Assay kits (ELISA, Diagnostic System Laboratories, TX, USA), specific for IGF-1 and IGFBP-3 respectively. The kit has a coefficient of variation for intra assay reproducibility of less than 10%. By testing duplicate samples, we estimated the average errors across 18 observations to be about 0.8 ng/ml for serum IGF-1 and about 15 ng/ml for IGFBP-3.

All the radiographs were assessed by two examiners at two different occasions at an interval of at least a week to check the intraexaminer and interexaminer reliability. Statistical analysis was performed with SPSS software. Analysis of variance test was used to compare mean IGF-1 and IGFBP-3 corresponding to six stages of cervical vertebrae maturation stages. Linear Pearson’s correlations were performed to determine the trends of IGF-1, IGFBP-3, and its ratio relating to CVM stage. The kappa statistic was used to measure inter and intra examiner reliability. *P* value <0.05 was considered as statistically significant.

## Results

**Graph 1:**
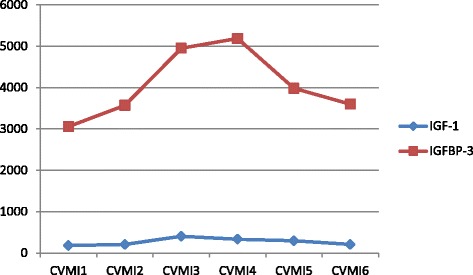
Mean Serum levels of IGF-1 and IGFBP-3 at each CVMI stage

The mean values and standard deviations of IGF-1 and IGFBP-3 serum levels in 90 female subjects are shown in Table 1 and represented graphically in Graph 1.

**Table 1:** Means and standard deviations of serum IGF-1, IGFBP-3 and their ratio at each CVMI stage

CVMI Stage	*n*	Mean Age (years)	IGF-1 (ng/ml)		IGFBP-3 (ng/ml)		Mean IGF-1/IGFBP-3 ratio
			Mean	SD	Mean	SD	
1 (Initiation)	15	8.5	180.9	15.9	3056	1087.6	0.066
2 (Acceleration)	15	9.3	208.4	10.2	3572.1	1209.4	0.064
3 (Transition)	15	11.5	403.3	12.3	4952.9	1169.8	0.084
4 (Deceleration)	15	14.4	332.4	12.8	5186.8	1384.2	0.068
5 (Maturation)	15	16.1	294.5	10.9	3984.1	1176.2	0.079
6 (Completion)	15	19.2	208.4	10	3599.5	1113.8	0.062

The kappa values for inter and intra examiner reliability were 0.82 and 0.94, respectively.

A steady increase was noted in serum IGF-1 levels from CVMI1 to CVMI2 with a mean value of 208.4 ± 10.2 ng/ml. Further a sharp increase was observed from CVMI2 to CVMI3 with a mean value of 403.3 ± 12.3 ng/ml. A progressive decline in mean serum IGF-1 levels was observed from CVMI3 to CVMI6. It was observed that mean serum IGF-1 levels at CVMI6 or at skeletal stage of growth completion was relatively high (208.4 ± 10 ng/ml) than at CVMI1.

The data showed that mean serum IGFBP-3 levels were lowest at CVMI1 stage of cervical development, with mean value of 3056 ± 1087.6 ng/ml. Further there was a gradual rise from CVMI1 to CVMI2, with mean value of 3572.1 ± 1209.4 ng/ml. Thereafter, mean serum IGFBP-3 levels showed a sharp rise from CVMI2 to CVMI3, with mean value of 4952.9 ± 1169.8 ng/ml. Further rise was seen from CVMI3 to CVMI4 where highest levels were found (5186.8 ± 1384.2 ng/ml). Further, a sharp decline was seen from CVMI4 to CVMI5, with mean levels of 3984.1 ± 1176.2 ng/ml. Finally the levels showed a further decrease with lowest value observed in CVMI6 (3599.5 ± 1113.8 ng/ml). However, the mean levels at CVMI6 were found to be relatively higher than that at CVMI1 stage of cervical development. The data also revealed high standard deviations of mean serum IGFBP-3 levels for each skeletal maturity stage.

One-way ANOVA (analysis of variance) test revealed a highly significant difference of means for IGF-1 (*P* < 0.001), IGFBP-3 and their ratio (*P* < 0.05) between and within the cervical (CVMI) stages as shown in Table 2.

**Table 2:** 1-way ANOVA showing IGF-1, IGFBP-3 and their ratio differences’ between and within the CVMI stages

		Sum of squares	df	Mean square	*F*	Sig.
Serum IGF-1 (ng/ml)	Between Groups	566437.3	5	113287.4	757.07	0.000(**)
	Within Groups	12569.5	84	149.6		
	Total	579006.9	89			
Serum IGFBP-3 (ng/ml)	Between Groups	5.29	5	1.05	7.42	0.000(**)
	Within Groups	1.19	84	1425822.1		
	Total	1.72	89			
IGF-1/IGFBP-3 Ratio	Between Groups	0.006	5	0.001	2.77	0.023(*)
	Within Groups	0.037	84	0.000		
	Total	0.043	89			

(*) Significant (*P* < 0.05)

(**) Highly significant (*P* < 0.01, *P* < 0.001)

Post hoc analysis (least significant difference) revealed that the IGF-1 serum levels at CVMI3 stage were statistically significantly higher than at the CVMI1, CVMI2, CVMI4, CVMI5 and CVMI6 at *P* < 0.001 as shown in Table 3.

**Table 3:** The Post-hoc analysis (least significant difference) comparing IGF-1 serum levels for each of the 6 CVMI stages

CVMI Stage	CVMI Stage	Mean Difference	Significance
1	2	−27.5^*^	0.000(**)
	3	−222.3^*^	0.000(**)
	4	−151.5^*^	0.000(**)
	5	−113.6^*^	0.000(**)
	6	−27.5^*^	0.000(**)
2	1	27.5^*^	0.000(**)
	3	−194.8^*^	0.000(**)
	4	−123.9^*^	0.000(**)
	5	−86.07^*^	0.000(**)
	6	−.02	0.996(NS)
3	1	222.3^*^	0.000(**)
	2	194.8^*^	0.000(**)
	4	70.8^*^	0.000(**)
	5	108.7^*^	0.000(**)
	6	194.8^*^	0.000(**)
4	1	151.5^*^	0.000(**)
	2	123.9^*^	0.000(**)
	3	−70.8^*^	0.000(**)
	5	37.9^*^	0.000(**)
	6	123.9^*^	0.000(**)
5	1	113.6^*^	0.000(**)
	2	86.07^*^	0.000(**)
	3	−108.7^*^	0.000(**)
	4	−37.9^*^	0.000(**)
	6	86.04^*^	0.000(**)
6	1	27.5^*^	0.000(**)
	2	0.02	0.996(NS)
	3	−194.8^*^	0.000(**)
	4	−123.9^*^	0.000(**)
	5	−86.04^*^	0.000(**)

(**) Highly significant (*P* < 0.01, *P* < 0.001),(*) Significant (*P* < 0.05), NS (Non significant)

Post hoc analysis (least significant difference) revealed that the IGFBP-3 serum levels at CVMI3 stage were statistically significantly higher than at the CVMI1, CVMI2, CVMI5 and CVMI6 at *P* < 0.001 as shown in Table 4.

**Table 4:** The Post-hoc analysis (least significant difference) comparing IGFBP-3 serum levels for each of the 6 CVMI stages

CVMI Stage	CVMI stage	Mean Difference	Significance
1	2	-516.1	0.240(NS)
	3	-1896.9^*^	0.000(**)
	4	-2130.8^*^	0.000(**)
	5	-928.1^*^	0.036(*)
	6	-543.5	0.216(NS)
2	1	516.1	0.240(NS)
	3	-1380.8^*^	0.002(**)
	4	-1614.6^*^	0.000(**)
	5	-411.9	0.347(NS)
	6	-27.4	0.950(NS)
3	1	1896.9^*^	0.000(**)
	2	1380.8^*^	0.002(**)
	4	-233.8	0.593(NS)
	5	968.8^*^	0.029(**)
	6	1353.4^*^	0.003(**)
4	1	2130.8^*^	0.000(**)
	2	1614.6^*^	0.000(**)
	3	233.8	0.593(NS)
	5	1202.7^*^	0.007(**)
	6	1587.2^*^	0.000(**)
5	1	928.1^*^	0.036(*)
	2	411.9	0.347(NS)
	3	-968.8^*^	0.029(*)
	4	-1202.7^*^	0.007(**)
	6	384.5	0.380(NS)
6	1	543.5	0.216(NS)
	2	27.4	0.950(NS)
	3	-1353.4^*^	0.003(**)
	4	-1587.2^*^	0.000(**)
	5	-384.5	0.380(NS)

(**) Highly significant (*P* < 0.01, *P* < 0.001), ﻿(*)﻿Significant (*P* < 0.05)

NS Not Significant

Linear Pearson’s correlations were performed to determine the trends of serum IGF-1 levels relating to the cervical skeletal maturational stages. A high positive correlation of +0.91 was observed between serum IGF-1 levels from CVMI1 to CVMI3 and a high negative correlation of −0.97 was observed from CVMI4 to CVMI6. A significant positive correlation of 0.96 (*P* < 0.05) was observed between IGFBP-3 levels and skeletal maturity stages from CVMI1 to CVMI3. A highly significant negative correlation (*r* = −1) (*P* < 0.001) was also observed from CVMI4 to CVMI6.

Linear Pearson’s correlations were also performed to determine the correlation of IGF-1, IGFBP-3, and their ratio with each other biochemical parameter, shown in Table 5.

**Table 5:** Pearson’s Correlations of IGF-1, IGFBP-3 and their ratio

		Serum IGF-1 (ng/ml)	Serum IGFBP-3 (ng/ml)	IGF-1/IGFBP-3 Ratio
Serum IGF-1 (ng/ml)	Pearson Correlation	1	0.529^**^	0.297^**^
	Sig. (2-tailed)		0.0	0.005
	N	90	90	90
Serum IGFBP-3 (ng/ml)	Pearson Correlation	0.529^**^	1	-0.609^**^
	Sig. (2-tailed)	0.0		0.0
	N	90	90	90
IGF-1/IGFBP-3 Ratio	Pearson Correlation	0.297^**^	-0.609^**^	1
	Sig. (2-tailed)	0.005	0.0	
	N	90	90	90

**. Correlation is significant at the 0.01 level (2-tailed)

The data showed that all the three parameters had a statistically significant correlation with each other at *P* < 0.01. Also, a negative correlation was observed between serum IGFBP-3 and ratio between IGF-1 and IGFBP-3 with correlation coefficient of −0.609, statistically significant at *P* < 0.001.

## Discussion

The accurate determination of a child’s maturational status and the need of a potential biochemical maturity indicator cannot be overemphasized. During pubertal growth spurt, the bones of face and jaw also undergo a number of growth changes along with the rapid increase in rate of linear growth at that time [4, 26–28]. To the clinical orthodontist, this period of the adolescent growth spurt represents the most desirable time for orthopaedic and orthodontic therapy.

Skeletal age has probably been the most commonly used index of biologic maturity, assessed by a radiograph of one or more areas of the body. Hand wrist radiograph has long been used as a method of choice for assessment of skeletal maturity. Because of the complexity in identification of landmarks, staging subjectivity and additional radiation exposure, its use is now discouraged. Use of CVMI staging does not require an additional radiation exposure in orthodontic patients but assessment of maturation status depends on the subjective evaluation and perception of the observer, thereby questioning the repeatability and validity [20].

Hence, there was a need to determine whether serum levels of IGF-1 and their binding protein (IGFBP-3) would serve as a useful marker for the orthodontist in assessing maturation level and growth clinically. Also it was important to evaluate whether serum levels of IGF-1 and IGFBP-3 should be investigated longitudinally in relation to facial growth. It may become an aid for the orthodontist or may simply confirm the value of a careful growth assessment. In either case, the data would have been useful.

This study comprised of only female subjects as IGF-1 and IGFBP-3 serum levels differ in males and females [29]. As a result, any confounding gender bias is eliminated from the study and results may help in forming the basis of establishing reference ranges of serum IGF-1 and IGFBP-3 in female population.

A major problem in IGF-1 measurement is the interference of IGFBPs in the assay. Direct determinations in untreated serum samples give false values because of the extremely slow dissociation of the IGF-1/IGFBP-3 complexes during the assay incubation. However, various techniques have been applied to physically separate IGF-1 from its binding proteins before measurement. Furthermore, the remaining IGFBPs may still interfere in the assay. Studies showed that IGF-1 radioimmunoassays (RIAs) do not quantitatively measure the IGF-1 content of serum [30]. To avoid these difficulties, ELISA method was used in which special sample preparation is not required before measurement; therefore, no predilution is done which leads to increased sensitivity of the assay procedure.

Results of this study showed that IGF-1 levels were lowest at initiation stage of cervical development (Table 1). Thereafter a steady increase was noted from CVMI1 to CVMI2. Further a sharp increase was observed from CVMI2 to CVMI3 with a mean value of 403.3 ± 12.3 ng/ml at CVMI3. The observed peak levels at CVMI3 (transition stage of cervical development) coincide with stage of maximum facial growth as shown by studies of Hassel and Farman [17]. Hence, the results of this study revealed highest serum IGF-1 levels at cervical stages that correspond to greatest amount of facial growth. However, this finding is in contrast with the findings of previous study by Masoud et al. [20] that showed IGF-1 levels to peak at maturation stage (CVMI5) of cervical development. This finding could be attributed to the fact that only female subjects were investigated in our study. Also, a low standard deviation was observed in mean serum IGF-1 levels (Table 1) at each skeletal maturity stage which depicts that not much of individual variation is observed in each stage of cervical development. This finding again is in contrast with previous study by Masoud et al. [20] who noted wide individual variation in the IGF-1 levels at each cervical stage. This observation can be related to the larger sample size with equal distribution of subjects in each group of skeletal maturity stages in our study to maintain the purity of sample. Another possible explanation for the difference observed in stages of peak rise in IGF-1 levels in the two studies could be related to the fact that the two data are from two different background populations. Although the sequence of pubertal changes in adolescence is predictable, the timing of puberty is variable. Variations in pubertal timing depend on genetic and environmental factors [31]. In addition, secular trend appears to influence the physiological range in the timing of pubertal onset [32].

The post-hoc test (Table 3) reveals a statistically significant difference in IGF-1 levels between all stages (except between CVMI2 and CVMI6), indicate that IGF-1 levels were significantly different at different stages of skeletal maturity and thus may have a specific range for specific skeletal stage. The data, therefore, could also be used to as a guideline to establish a reference range of serum IGF-1 levels in North Indian population. Further, the results of our study appear to be reliable as indicated by observed low standard deviation of mean serum IGF-1 values at each skeletal maturity stage.

A marked positive correlation, with correlation coefficient of +0.91, was observed between serum IGF-1 levels from CVMI1 to CVMI3. This correlation might be directly translated to relationship between IGF-1 levels and maxillary and mandibular growth since previous studies by Ohlsson et al have shown that growth hormone (GH) therapy accelerate cartilage growth [33].

Levels of serum IGFBP-3 were lowest at CVMI1 stage of cervical development with mean values of 3056 ± 1087.6 ng/ml (Table 1). Thereafter, serum IGFBP-3 levels showed a sharp rise from CVMI2 o CVMI3 with a mean value of 4952.9 ± 1169.8 ng/ml at CVMI3, the difference between the two stages being statistically significant. Further rise was seen from CVMI3 to CVMI4 where highest levels were seen with mean value of 5186.8 ± 1384.2 ng/ml at CVMI4. This is in marked contrast with the developmental pattern of IGF-1 we observed. However, the difference in mean levels at CVMI3 and CVMI4 were found to be statistically non-significant. Also, Pearson’s linear correlations revealed a marked positive correlation (*r* = 0.96) (*P* < 0.05) between IGFBP-3 levels and skeletal maturity stages from CVMI1 to CVMI4 and a marked negative correlation (*r* = −1) (*P* < 0.001) from CVMI5 to CVMI6. Further a statistically significant correlation (*r* = 0.529) (*P* < 0.001) was observed between serum IGF-1 and IGFBP-3 values (Table 5). This indicates that IGFBP-3 can also serve as potential biochemical indicator of adolescent growth spurt and can be speculated as a potential biochemical marker for assessment of skeletal maturity, in a way similar to IGF-1. However, wide individual variations were noted in serum IGFBP-3 levels in this study. Using longitudinal data, Bushang et al showed substantial variation in condylar growth in children between 6 to 16 years of age [34]. Variation in IGFBP-3 levels observed might account for this and also for individual variation in yearly increment of mandibular growth.

A marked positive correlation (*r* = 0.96) (*P* < 0.05) was observed between IGFBP-3 levels and skeletal maturity stages from CVMI1 to CVMI3. This correlation might be directly translated to relationships between IGFBP-3 and mandibular growth as a statistically significant correlation (*r* = 0.52) (*P* < 0.001) was found between IGF-1 and IGFBP-3 (Table 5).

A comparison of IGF-1 and IGFBP-3 serum concentrations revealed that they did not exhibit the same developmental pattern at various skeletal maturity stages. Rate of increase in IGF-1 levels through various CVMI stages was greater than the increase in IGFBP-3, leading to an increased molar ratio between IGF-1 and IGFBP-3 at CVMI3, when growth velocity is high. The mean ratio was found to be highest (0.084) at CVMI3 or transition stage of cervical development (Table 6). The ratio at CVMI3 stage was found to be statistically higher than those at CVMI1 and CVMI4 at *P* < 0.05 and CVMI2 and CVMI6 at *P* < 0.01 (Table 6).

**Table 6:** Post-hoc analysis (least significant difference) comparing ratio of serum IGF-1 and IGFBP-3 for each of the six CVMI stages

CVMI Stage	CVMI stage	Mean difference	Significance
1	2	.001	0.8(NS)
	3	−.018^*^	0.01(NS)
	4	−.001	0.8(NS)
	5	−.012	0.09(NS)
	6	.003	0.6(NS)
2	1	−.001	0.8(NS)
	3	−.020^*^	0.009(**)
	4	−.003	0.6(NS)
	5	−.014	0.05(NS)
	6	.001	0.8(NS)
3	1	.018^*^	0.016(*)
	2	.020^*^	0.009(**)
	4	.016^*^	0.029(*)
	5	.005	0.4(NS)
	6	.022^*^	0.005(**)
4	1	.001	0.8(NS)
	2	.003	0.6(NS)
	3	−.016^*^	0.029(*)
	5	−.011	0.1(NS)
	6	.005	0.4(NS)
5	1	.012	0.09(NS)
	2	.014	0.05(NS)
	3	−.005	0.4(NS)
	4	.011	0.1(NS)
	6	.016^*^	0.035(*)
6	1	−.003	0.6(NS)
	2	−.001	0.8(NS)
	3	−.022^*^	0.005(**)
	4	−.005	0.4(NS)
	5	−.016^*^	0.035(*)

(*) Significant (*P* < 0.05)

(**) Highly significant (*P* < 0.01, *P* < 0.001)

NS not Significant

Also, a negative correlation was observed between serum IGFBP-3 and ratio between IGF-1 and IGFBP-3 with correlation coefficient of −0.609, statistically significant at *P* < 0.001, indicating that relatively a steeper rise was observed in serum IGF-1 levels but not in IGFBP-3 levels such that the ratio between the two increases from CVMI2 to CVMI3. Thus, the ratio between IGF-1 and IGFBP-3 can also be speculated to have a potential role in assessment of pubertal growth spurt.

## Conclusions

1. Mean serum IGF-1 levels were found to be highest at CVMI3 stage of CVMI 403.3 ± 12.3 ng/ml.
2. The post-hoc test revealed a significant difference in IGF-1 levels between all stages of CVMI, thereby indicating a specific range of IGF-1 levels for a specific skeletal stage.
3. Mean serum IGFBP-3 levels were found to be highest at CVMI4 stage of CVMI with a mean value of 5186.8 ± 1384.2 ng/ml.
4. The mean serum IGFBP-3 levels at CVMI4 were found to be significantly higher than the levels at all other CVMI stages except CVMI3 stage. The difference in mean serum levels at CVMI3 and CVMI4 was statistically non-significant.
5. A statistically significant coefficient of correlation observed between serum IGF-1 and IGFBP-3 values indicate that IGFBP-3 can also serve as potential biochemical indicator for assessment of skeletal maturity.
6. The ratio between IGF-1 and IGFBP-3 at CVMI3 stage was found to be significantly higher than those at CVMI1 and CVMI4 at *p* < 0.05 and CVMI2 and CVMI6 at *p* < 0.01. The ratio can also have a potential role in assessment of pubertal growth spurt.

It is proposed that longitudinal studies are needed to confirm the usefulness of IGF-1 and IGFBP-3 to accurately determine the timing, and possibly the intensity, of a patient’s growth spurt and to determine whether they are good predictors of residual facial growth. Further, it could also help to establish population specific reference ranges of serum IGF-1 and IGFBP-3 at different skeletal maturity stages thus reducing the need for sequential radiographic exposure for assessment of maturation level of the individuals.

## References

[1] Fishman L, Subtenly J (2000). Maturational development and facial form relative to treatment timing. Early orthodontic treatment.

[2] Anderson DL, Thompson GW, Popovich F (1975). Interrelationship of dental maturity, skeletal maturity, height and weight from age 4-14 years. Growth.

[3] Marshall WA, Tanner JM, Falkner F (1986). Puberty. Human growth.

[4] Bambha J (1961). Longitudinal cephalometric roentgenographic study of the face and craniun in relation to body height. J Am Dent Assoc.

[5] Johnston F, Hufham HJ, Moreschi A, Terry G (1965). Skeletal maturation and cephalometric development. Angle Orthod.

[6] Krogman WM (1979). Maturation age of the growing child in relation to the timing of statural and facial growth at puberty. Trans Stud Coll Physicians Phila.

[7] Chertkow S (1980). Tooth mineralization as an indication of the pubertal growth spurt. Am J Orthod.

[8] Demirjian A, Buschang PH, Tanguay R, Patterson DK (1985). Interrelationships among measure of somatic, skeletal, dental, and sexual maturity. Am J Orthod.

[9] Fishman LS (1987). Maturation patterns and prediction during adolescence. Angle Orthod.

[10] Tanner JM, Tanner JM (1986). Use and abuse of growth standards. Human growth.

[11] Fishman LS (1982). Radiographic evaluation of skeletal maturation. A clinically oriented method based on hand-wrist films. Angle Orthod.

[12] Greulich WW, Pyle SI (1959). Radiographic atlas of the skeletal development of the hand and wrist.

[13] Tanner JM, Whitehouse RH, Cameron N, Marshall WA, Healy MJR, Goldstein H (1975). Assessment of skeletal maturity and prediction of adult height (TW2 method).

[14] Lamparski DG (1972). Skeletal age assessment utilizing cervical vertebrae [thesis].

[15] O’Reilly M, Yanniello GJ (1988). Mandibular growth changes and maturation of the cervical vertebrae—a longitudinal cephalometric study. Angle Orthod.

[16] Hassel B, Farman AG (1995). Skeletal maturation evaluation using cervical vertebrae. Am J Orthod Dentofacial Orthop.

[17] Kucukkeles N, Acar A, Biren D, Arun T (1999). Comparisons between cervical vertebrae and hand-wrist maturation for the assessment of skeletal maturity. J Clin Pediatr Dent.

[18] Baccetti T, Franchi L, McNamara JA (2005). The cervical vertebral maturation (CVM) method for the assessment of optimal treatment timing in dentofacial orthopediCVMI. Sem in Orthod.

[19] Masoud M, Masoud I, Kent RL, Gowharji N, Cohen LE (2008). Assessing skeletal maturity by using blood spot insulin-like growth factor I (IGF-1) testing. Am J Orthod Dentofacial Orthop.

[20] Finkelstein JW, Roffwarg HP, Boyar RM, Krean J, Hellman L (1972). Age related change in 24 hour spontaneous secretion of growth hormone. JClin Endocrinol Metab.

[21] Loche S, Casini MR, Faedda A (1996). The GH/IGF-1 axis in puberty. Br J Clin Pract Suppl.

[22] Ishaq RA, Soliman SA, Foda MY, Fayed MM (2012). Insulin-like growth factor I: a biologic maturation indicator. Am J Orthod Dentofacial Orthop.

[23] Masoud M, Masoud I, Kent RL, Gowharji N, Cohen LE (2009). Relationship between blood-spot insulin-like growth factor 1 levels and hand-wrist assessment of skeletal maturity. Am J Orthod Dentofacial Orthop.

[24] Juul A, Dalgaard P, Blum WF, Bang P, Hall K, Michaelsen KF, Muller J, Skakkebaek NE (1995). Serum levels of insulin-like growth factor (IGF)-binding protein-3 (IGFBP-3) in healthy infants, children, and adolescents: the relation to IGF-1, IGF-1I, IGFBP-1, IGFBP-2, age, sex, body mass index, and pubertal maturation. J Clin Endocrinol Metab.

[25] Blum WF, Cotterill AM, Postel-Vinay MC, Ranke MB, Savage MO, Wilton P (1994). Improvement of diagnostic criteria in growth hormone insensitivity syndrome: solutions and pitfalls. Pharmacia Study Group on Insulin-like Growth Factor I Treatment in Growth Hormone Insensitivity Syndromes. Acta Paediatr Suppl.

[26] Nanda RS (1955). The rates of growth of several facial components measured from serial cephalometric roentgenograms. Am J Orthod.

[27] Hunter CJ (1966). The correlation of facial growth with body height and skeletal maturation at adolescence. Angle Orthod.

[28] Seckel HPG (1950). Six examples of precocious sexual development. Am J Dis Child.

[29] Juul A, Bang P, Hertal NT, Main K, Dalgaard P, Jorgensen K (1999). Serum insulin –like growth factor-I in1030 healthy children, adolescence and adults: relation to age, sex, stage of puberty, testicular size and body mass index. J Clin Endocrinol Metab.

[30] Daughaday WH, Mariz IK, Blethen SL (1980). Inhibition of access of bound somatomedin to membrane receptor and immunobinding sites: a comparison of radioreceptor and radioimmunoassay of somatomedin in native and acid-ethanol-extracted serum. J Clin Endocrinol Metab.

[31] Floyd B (2000). Can socioeconomic factors account for “atypical” correlations between timing, peak velocity, and intensity of adolescent growth in Taiwanese girls?. Am J Hum Biol.

[32] Floyd B (2003). Patrilineal family values, family planning and variation in stature among Taiwanese six-year-olds. J Biosoc Sci.

[33] Ohlsson C, Bengtsson BA, Isaksson GP (1998). Growth hormone and bone. Endocrinol.

[34] Buschang PH, Santos-Pinto A, Demirjian A (1999). Incremental growth charts for condylar growth between 6 and 16 years of age. Eur J Orthod.

